# Interfacial Stress Analysis of Adhesively Bonded Lap Joint

**DOI:** 10.3390/ma12152403

**Published:** 2019-07-28

**Authors:** Shiuh-Chuan Her, Cheng-Feng Chan

**Affiliations:** Department of Mechanical Engineering, Yuan Ze University, Taoyuan City 320, Taiwan

**Keywords:** double lap joint, elasticity theory, shear stress, peel stress

## Abstract

The use of adhesively bonded joints in place of traditional joining techniques such as bolted or rivet joints is becoming greatly popular in recent years. Interfacial stress in the adhesive is critical to the strength of adhesively bonded joints. It is necessary to predict the interfacial stresses accurately to ensure the safety of joints. In this work, an analytical model is explicitly presented to evaluate the stresses in a double lap joint. The equilibrium equations in the adhesive overlap region are derived on the basis of elasticity theory. The governing equations are presented in terms of shear and peel stresses in the adhesive. Analytical solutions are derived for the shear and peel stresses, which are considered to be the main reason for the failure of the double lap joint. To verify the analytical solutions, the finite element method is conducted using the commercial package ANSYS. Results from the analytical solution agree well with finite element results and numerical investigations available in the literature. The effect of the adhesive thickness, shear modulus, adherend Young’s modulus and bonding length on the shear and peel stresses in the adhesive of the double lap joint are studied. Numerical results demonstrate that both the maximum shear and peel stress occur at both ends of the bonding region. The maximum values of the shear and peel stresses increase as the adhesive thickness decreases and as the adhesive shear modulus increases provided that the adhesive thickness is sufficiently small. The simplicity and capability to obtain analytical expressions of the shear and peel stresses for double lap adhesive bonded joints makes the proposed analytical model applicable for the stress analysis and preliminary structural design.

## 1. Introduction

Adhesive joints have been widely used in a variety of engineering applications, in particular for the sectors where bonding is critical to the safety of structures, e.g., automotive and aerospace industries [1]. It offers some advantages such as more uniform stress distribution, reduction of stress concentration, less weight and easy to be fabricated in comparison with welding, bolting and fastening [2]. Single lap joint, double lap joints and scarf joints are the most common bonded joints [3]. Among them, the single lap joint is the most generally studied owing to its simple geometry and easy to prepare, although it exhibits the worst specific strength in these three different joints configurations [4]. The poor performance can be attributed to the eccentricity of the applied loads resulting in a bending moment on the joint, which induces the high stress concentrations at the ends of the bonding region [5]. The performance of the double lap joint is improved by reducing the stress concentration of both peel and shear stresses. The reduction of peel stress is due to the elimination of the eccentricities of applied loads, while shear stress is reduced by removing the differential straining effect [6]. The scarf joint is considered as the best in terms of strength for the same bonding region, by further reducing the stress concentration because of the elimination of the geometry discontinuity, which appears in the lap joints [7]. Although, adhesively bonded joints have been used more often than that of mechanical joints in connecting the structural components owing to their advantages such as more uniform interfacial stress distributions, capability of joining different materials and high resistance to fatigue failure [8,9]. However, in the adhesively bonded joints, high stress concentration occurs at both ends of the bonding region, which severely affects the strength of the joint. In order to employ the adhesive bonding technique appropriately and to improve the load carrying capacity of the joint, interfacial stresses distributions in the adhesive should be determined accurately [10].

A variety of techniques have been proposed for adhesively bonded joints, either analytical, numerical or experimental. There are several analytical models available for adhesively bonded joints. Volkersen [11] reported a simple model for the single lap joint, which assumed shear stress only in the adhesive, while adherends were subjected to longitudinal normal stress. Volkersen’s model is considered as one of the most important and pioneer contributions to the adhesively bonded joints. Goland and Reissner [12] revised Volkersen’s model by introducing peel stresses and large deflection to the adherends. Later, Hart-Smith [13] proposed a model, which modified the Goland and Reissner model with plastic deformation in the adhesive. Klarbring and Movchan [14] proposed an asymptotic modeling for adhesive joints in a thin compound beam with a layered structure. Raous et al. [15] presented a model coupling adhesion, Coulomb friction and unilateral contact. In their model, adhesion was characterized by an internal valuable to represent the intensity of adhesion. Yousefsani and Tahani [16] used full layerwise theory to predict the shear stress, peeling stress and von Mises stress in the adhesive layer for adhesively bonded joints subjected to uniaxial tension and bending moment. Wu and Zhao [17] proposed stress functions of the interfacial shear and peeling stresses for the adhesively bonded joint, then employed the variational method to determine the interfacial stresses using the theory of minimum complimentary strain energy. Fernlund [18] incorporated the theory of fracture mechanics with energy balance to derive analytical solutions for the maximum shear and peeling stresses at the ends of the overlap region for straight and curved lap joints. Oplinger [19] presented an alternative model, which included bending deflections of both the adherend and adhesive in the overlap region of the single lap joint. Tsai et al. [20] derived an analytical solution based on the assumption of linear shear stress distribution across the adherend thickness. Her [21] reported theoretical solutions for the shear stress in the adhesive and longitudinal normal stress in the adherends using the elasticity theory. Recently, a rapid increase of computational capability has significantly attracted a lot of attention in numerical simulation as an accurate and effective technique, in particular for the finite element method. Extensive review of finite element-based techniques is provided by He [22]. Mokhtari et al. [23] used a 3-D finite element commercial code ABAQUS to investigate the influences of material properties including ply thickness, Young’s modulus and orientations on the stress distributions in the composite double lap joint. Stuparu et al. [24] employed the eXtended finite element method (XFEM) using cohesive zone modelling (CZM) to evaluate the failure of a single-lap joint that adhesively bonded two different materials. Moya-Sanz et al. [25] developed a 2-D numerical model in the Abaqus/Standard to investigate the effects of geometry such as recessing and chamfering of the adherends and adhesive on the failure load of a single-lap joint under uniaxial tensile force, using the cohesive zone model. Hou et al. [26] proposed a novel concept to reduce the stress concentration at both ends of the bonding region in a double lap joint by making a slot in the inner adherend using finite element numerical simulation. Santos et al. [3] employed a numerical method based on XFEM to predict the stress distribution, strength and damage propagation in the adhesively bonded joints. Campilho and Fernandes [2] used the finite element method and cohesive zone model to investigate the performance of single lap joints with different adhesives. Panigrahi and Pradhan [27] conducted the 3-D nonlinear finite element analysis to study the initiation and growth of the delamination in the adhesive of a composite double lap joint. Several researchers have conducted experimental tests to determine the strength and failure load of adhesively bonded joints. Khan et al. [28] performed experimental tests to investigate the influence of the adherend layup, adhesive material property and thickness on the strength of double-lap joint according to ASTM D3528-96 specifications. Ozel et al. [29] experimentally studied the failure load of a single-lap joint with different adherends. Tsai and Morton [30] employed the full-field moiré interferometry to examine in-plane deformations of the adhesive in a double-lap joint. Neto et al. [31] conducted experimental tests on the adhesively bonded composite joints with brittle and ductile adhesives using a cohesive zone model to determine the joint strength. Akhavan-Safar et al. [32] experimentally investigated the effect of adhesive thickness on the strength of adhesively bonded single lap joints. Gultekin et al. [33] studied the effect of the adherend width on the strength of an adhesively bonded single lap joint experimentally.

It is essential to understand the stresses acting on an adhesively bonded lap joint to determine whether the structure is safe or failure under the normal operation. Although, in these days, stresses in the adhesively bonded joints can be completely calculated using the finite element method, the need for analytical solutions still exists. Closed-form solutions can provide a better understanding of a phenomenon than that of numerical results. Furthermore, formulae can be of a simple and quick tool to engineers for a preliminary design of joints. Present work studies the double lap joint, and focus on the interfacial stresses including the shear and peel stresses in the adhesive layer. The goal is to provide analytical solutions to predict the shear and peel stresses based on the theory of elasticity. The analytical predictions are validated with the finite element results. The effects of the bonding length, adhesive thickness and elastic moduli of the adherend and adhesive on the shear and peel stresses are investigated through a parametric study.

## 2. Stress Analysis of Double Lap Joint

**Figure 1:**
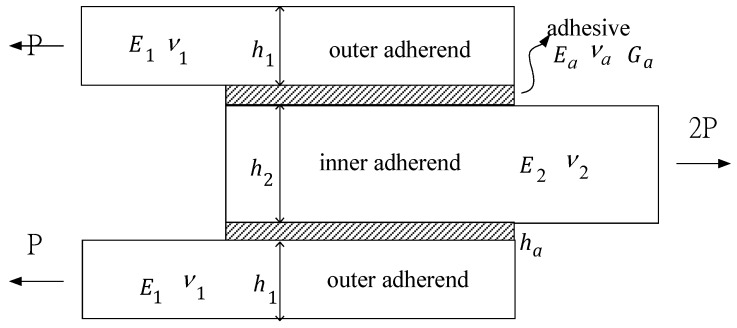
Geometric model for an adhesive double lap joint.

**Figure 2:**
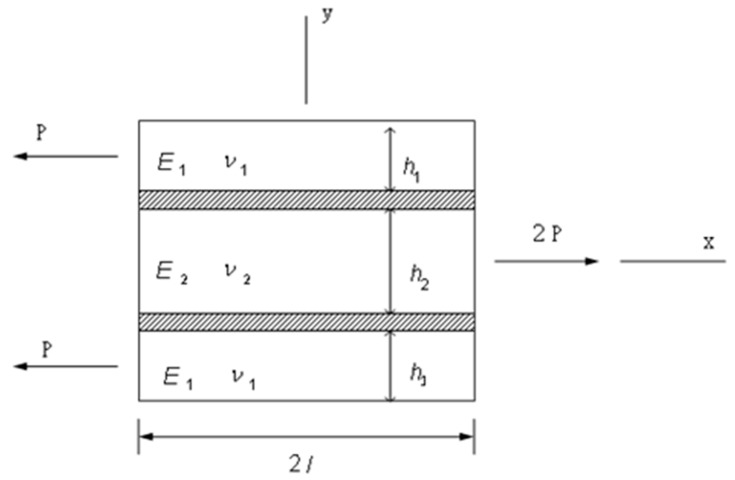
Geometric model in the overlap region.

**Figure 3:**
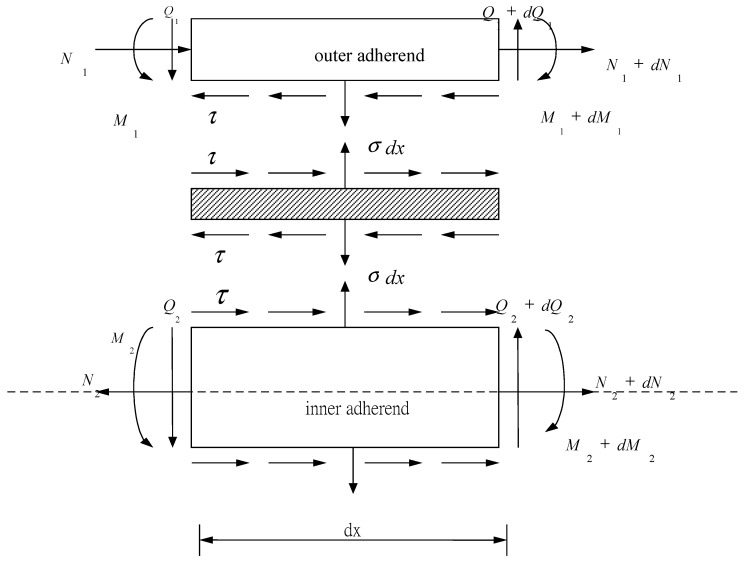
Free body diagram for an infinitesimal segment dx.

Double lap joints are commonly used in bonded joint construction since the peel stress in the adhesive is lower than that of the single lap joint. In addition, the bending effect which appears in the single lap joint can be avoided in a symmetrical double lap joint. However, the high stress concentration occurred at both ends of the bonding region is still the major concern for a double lap joint. In an adhesively bonded joint, the load can be smoothly transferred from one adherend to another via the adhesive layer in the bonding region, i.e., the adhesive acts as a medium for load transfer. The aim of this study is to derive analytical solutions to calculate the shear and peel stresses in the adhesive. The model presented in this work considers a double lap joint symmetrical with respect to its mid-plane as shown in Figure 1, where E, ν and *h* are the Young’s modulus, Poisson ratio and thickness, respectively, subscripts 1 and 2 denote the outer and inner adherends, respectively; $E_{a},\;G_{a},\;\nu_{a}$ and $h_{a}$ are the Young’s modulus, shear modulus, Poisson ratio and thickness of the adhesive, respectively. The two outer adherends are identical but can be of any thickness and material. Figure 2 shows the geometric model in the overlap region. The whole joint is in balance under the action of longitudinal forces. The two outer adherends are subjected to the same longitudinal force of P along *x*-axis, the inner adherend is subjected to an opposite load of intensity 2P. The free body diagram of an infinitesimal segment dx in the overlap region is shown in Figure 3, where σ and τ represent the peel and shear stresses in the adhesive, respectively; N, Q, M are the longitudinal force, transverse force and bending moment, respectively, subscripts 1 and 2 denote the outer and inner adherends, respectively.

The equilibrium of longitudinal force in the outer and inner adherends yields

(1a) $$\frac{dN_{1}}{dx}-\tau =0$$

(1b) $$\frac{dN_{2}}{dx}+2\tau =0$$

The equilibrium of the transverse force in the outer and inner adherends yields

(2a) $$\frac{dQ_{1}}{dx}-\sigma =0$$

(2b) $$\frac{dQ_{2}}{dx}=0$$

The equilibrium of the bending moment in the outer and inner adherends yields

(3a) $$\frac{dM_{1}}{dx}-Q_{1}+\frac{1}{2}\tau \;h_{1}=0$$

(3b) $$\frac{dM_{2}}{dx}-Q_{2}=0$$

The relationship between the transverse displacements $w_{i}$ and bending moments $M_{i}$ in the inner and outer adherends based on the Euler’s beam theory and plane strain condition can be written as

(4) $$\frac{d^{2}w_{i}}{dx^{2}}=-\frac{12(1-\nu^{2})}{E_{i}h_{i}^{3}}M_{i}\;(i\;=\;1,2)$$

The longitudinal displacements $u_{i}$ at the mid-plane of the inner and outer adherends can be relative to the longitudinal forces $N_{i}$ as follows

(5) $$\frac{du_{i}}{dx}=\frac{1-\nu_{i}^{2}}{E_{i}h_{i}}N_{i}\;(i\;=\;1,2)$$

Enforcing the displacement continuity at the interfaces between the adhesive and adherends, the longitudinal displacements at the top and bottom of the adhesive can be expressed in terms of the longitudinal and transverse displacements in the adherends as follows

(6) $$u_{a}^{top}=u_{1}+\frac{1}{2}h_{1}\frac{dw_{1}}{dx}\;u_{a}^{bottom}=u_{2}-\frac{1}{2}h_{2}\frac{dw_{2}}{dx}$$

where $u_{a}^{top}$,$u_{a}^{bottom}$ are the longitudinal displacements at the top and bottom of the adhesive layer.

The transverse strain $\varepsilon_{yya}$ and shear strain $\gamma_{xya}$ of the adhesive can be written as

(7a) $$\varepsilon_{yya}=\frac{w_{1}-w_{2}}{h_{a}}$$

(7b) $$\gamma_{xya}=\frac{u_{a}^{top}-u_{a}^{bottom}}{h_{a}}$$

The transverse strain $\varepsilon_{yya}$ and shear strain $\gamma_{xya}$ are relative to the peel stress σ and shear stress τ in the adhesive as follows

(8a) $$\varepsilon_{yya}=\frac{1-\nu_{a}^{2}}{E_{a}}\sigma$$

(8b) $$\gamma_{xya}=\frac{\tau }{G_{a}}$$

Substituting Equations (6) and (7b) into Equation (8b) then taking the derivative with respect to *x*, yields

(9) $$\frac{du_{1}}{dx}+\frac{1}{2}h_{1}\frac{d^{2}w_{1}}{dx^{2}}-\frac{du_{2}}{dx}+\frac{1}{2}h_{2}\frac{d^{2}w_{2}}{dx^{2}}=\frac{h_{a}}{G_{a}}\frac{d\tau }{dx}$$

Substituting Equations (4) and (5) into the above equation then taking the derivative with respect to *x*, yields

(10) $$\frac{1}{E_{1}^{\prime }h_{1}}\frac{dN_{1}}{dx}-\frac{1}{E_{2}^{\prime }h_{2}}\frac{dN_{2}}{dx}-\frac{6}{E_{1}^{\prime }h_{1}^{2}}\frac{dM_{1}}{dx}-\frac{6}{E_{2}^{\prime }h_{2}^{2}}\frac{dM_{2}}{dx}=\frac{h_{a}}{G_{a}}\frac{d^{2}\tau }{dx^{2}}$$

$$E_{i}^{\prime }=\frac{E_{i}}{1-\nu_{i}^{2}}\;(i\;=\;1,2);\;G_{a}=\frac{E_{a}}{2(1+\nu_{a})}$$

Substituting Equations (1) and (3) into the above equation then taking the derivative with respect to *x*, yields

(11) $$\frac{1}{E_{1}^{\prime }h_{1}}\frac{d\tau }{dx}+\frac{2}{E_{2}^{\prime }h_{2}}\frac{d\tau }{dx}-\frac{6}{E_{1}^{\prime }h_{1}^{2}}(\frac{dQ_{1}}{dx}-\frac{1}{2}\tau \;h_{1}\frac{d\tau }{dx})-\frac{6}{E_{2}^{\prime }h_{2}^{2}}(\frac{dQ_{2}}{dx}-\frac{1}{2}\tau \;h_{2}\frac{d\tau }{dx})=\frac{h_{a}}{G_{a}}\frac{d^{3}\tau }{dx^{3}}$$

Substituting Equation (2) into the above equation, leads to

(12) $$\frac{ha}{G_{a}}\frac{d^{3}\tau }{dx^{3}}-(\frac{4}{E_{1}^{\prime }h_{1}}+\frac{2}{E_{2}^{\prime }h_{2}})\frac{d\tau }{dx}+\frac{6}{E_{1}^{\prime }h_{1}^{2}}\sigma =0$$

Substituting Equation (7a) into Equation (8a) then taking the derivative with respect to *x* twice, yields

(13) $$\frac{h_{a}}{E_{a}^{\prime }}\frac{d^{2}\sigma }{dx^{2}}=\frac{d^{2}w_{1}}{dx^{2}}-\frac{d^{2}w_{2}}{dx^{2}}$$

$$E_{a}^{\prime }=\frac{E_{a}}{1-\nu_{a}^{2}}$$

Substituting Equation (4) into the above equation then taking the derivative with respect to *x*, leads to

(14) $$\frac{h_{a}}{E_{a}^{\prime }}\frac{d^{3}\sigma }{dx^{3}}=\frac{-12}{E_{1}^{\prime }h_{1}^{3}}\frac{dM_{1}}{dx}+\frac{12}{E_{2}^{\prime }h_{2}^{3}}\frac{dM_{2}}{dx}$$

Substituting Equation (3) into the above equation then taking the derivative with respect to *x*, leads to

(15) $$\frac{h_{a}}{E_{a}^{\prime }}\frac{d^{4}\sigma }{dx^{4}}=\frac{-12}{E_{1}^{\prime }h_{1}^{3}}(\frac{dQ_{1}}{dx}-\frac{h_{1}}{2}\frac{d\tau }{dx})+\frac{12}{E_{2}^{\prime }h_{2}^{3}}\frac{dQ_{2}}{dx}$$

Substituting Equation (2) into the above equation, yields

(16) $$\frac{h_{a}}{E_{a}^{\prime }}\frac{d^{4}\sigma }{dx^{4}}+\frac{12}{E_{1}^{\prime }h_{1}^{3}}\sigma -\frac{6}{E_{1}^{\prime }h_{1}^{2}}\frac{d\tau }{dx}=0$$

The peel stress is relative to the shear stress from Equation (12) as follows

(17) $$\sigma =[(\frac{4}{E_{1}^{\prime }h_{1}}+\frac{2}{E_{2}^{\prime }h_{2}})\frac{d\tau }{dx}-\frac{h_{a}}{G_{a}}\frac{d^{3}\tau }{dx^{3}}]/(\frac{6}{E_{1}^{\prime }h_{1}^{2}})$$

Substituting Equation (17) into Equation (16), leads to the governing differential equation in terms of the shear stress as follows

(18) $$\frac{d^{7}\tau }{dx^{7}}-\frac{G_{a}}{h_{a}}(\frac{4}{E_{1}^{\prime }h_{1}}+\frac{2}{E_{2}^{\prime }h_{2}})\frac{d^{5}\tau }{dx^{5}}+\frac{E_{a}^{\prime }}{h_{a}}\frac{12}{E_{1}^{\prime }h_{1}^{3}}\frac{d^{3}\tau }{dx^{3}}-[\frac{12E_{a}^{\prime }G_{a}}{E_{1}^{\prime 2}h_{1}^{4}h_{a}^{2}}+\frac{24E_{a}^{\prime }G_{a}}{E_{1}^{\prime }E_{2}^{\prime }h_{1}^{3}h_{2}h_{a}^{2}}]\frac{d\tau }{dx}=0$$

The characteristic equation for the above 7th order differential equation is

(19) $$m^{7}-\frac{G_{a}}{h_{a}}(\frac{4}{E_{1}^{\prime }h_{1}}+\frac{2}{E_{2}^{\prime }h_{2}})\;m^{5}+\frac{E_{a}^{\prime }}{h_{a}}\frac{12}{E_{1}^{\prime }h_{1}^{3}}m^{3}-[\frac{12E_{a}^{\prime }G_{a}}{E_{1}^{\prime 2}h_{1}^{4}h_{a}^{2}}+\frac{24E_{a}^{\prime }G_{a}}{E_{1}^{\prime }E_{2}^{\prime }h_{1}^{3}h_{2}h_{a}^{2}}]\;m=0$$

The roots of the above characteristic equations can be expressed as

$$m_{1}=0\;,\;\pm m_{2}\;,\;\pm m_{3}\;,\;\pm {\bar{m}}_{3}$$

where $m_{2}$ is real; $m_{3}$ and ${\bar{m}}_{3}$ are complex conjugate.

Thus, the solution of shear stress for the differential Equation (18) can be written as

(20) τ(x)=K1+K2sinh(m2)x+K3cosh(m2)x+K4sinhRe(m3)xcosIm(m3)x+K5sinhRe(m3)xsinIm(m3)x+K6coshRe(m3)xcosIm(m3)x+K7coshRe(m3)xsinIm(m3)x

where $K_{1},\;K_{2},\;K_{3},\;K_{4},\;K_{5},\;K_{6},\;K_{7}$ are constants to be determined by the boundary conditions.

Substituting Equation (20) into Equation (17), leads to the peel stress

(21) σ(x)=A1sinh(m2)x+A2cosh(m2)x+A3sinhRe(m3)xcosIm(m3)x+A4sinhRe(m3)xsinIm(m3)x+A5coshRe(m3)xcosIm(m3)x+A6coshRe(m3)xsinIm(m3)x

(22) $$A_{1}=[(\frac{4}{E_{1}^{\prime }h_{1}}+\frac{2}{E_{2}^{\prime }h_{2}})m_{2}K_{3}-\frac{h_{a}}{G_{a}}m_{2}^{3}K_{3}]/(\frac{6}{E_{1}^{\prime }h_{1}^{2}})$$

(23) $$A_{2}=[(\frac{4}{E_{1}^{\prime }h_{1}}+\frac{2}{E_{2}^{\prime }h_{2}})m_{2}K_{2}-\frac{h_{a}}{G_{a}}m_{2}^{3}K_{2}]/(\frac{6}{E_{1}^{\prime }h_{1}^{2}})$$

(24) A3=[(4E1′h1+2E2′h2)(Re(m3)K6+Im(m3)K5)−haGa(Re(m3)3K6−3Re(m3)Im(m3)2K6+3Re(m3)2Im(m3)K5−Im(m3)3K5)](6E1′h12)

(25) A4=[(4E1′h1+2E2′h2)(Re(m3)K7−Im(m3)K4)−haGa(Re(m3)3K7−3Re(m3)Im(m3)2K7−3Re(m3)2Im(m3)K4−Im(m3)3K4)](6E1′h12)

(26) A5=[(4E1′h1+2E2′h2)(Re(m3)K4+Im(m3)K7)−haGa(Re(m3)3K4−3Re(m3)Im(m3)2K4+3Re(m3)2Im(m3)K7−Im(m3)3K7)](6E1′h12)

(27) A6=[(4E1′h1+2E2′h2)(Re(m3)K5−Im(m3)K6)−haGa(Re(m3)3K5−3Re(m3)Im(m3)2K5−3Re(m3)2Im(m3)K6−Im(m3)3K6)](6E1′h12)

## 3. Boundary Conditions

The boundary conditions are enforced to determine the constants $K_{1}$~$K_{7}$ in Equation (20). Figure 2 shows the geometry of the bonding region. The boundary conditions can be expressed in terms of the longitudinal force *N*, transverse force *Q* and bending moment *M*, which act on the outer and inner adherends as follows.

(28a) $$x=l\;M_{1}=Q_{1}=N_{1}=0$$

(28b) $$M_{2}=Q_{2}=0$$

(28c) $$N_{2}=2P$$

(29a) $$x=-l\;M_{2}=Q_{2}=N_{2}=0$$

(29b) $$M_{1}=Q_{1}=0$$

(29c) $$N_{1}=P$$

**Figure 4:**
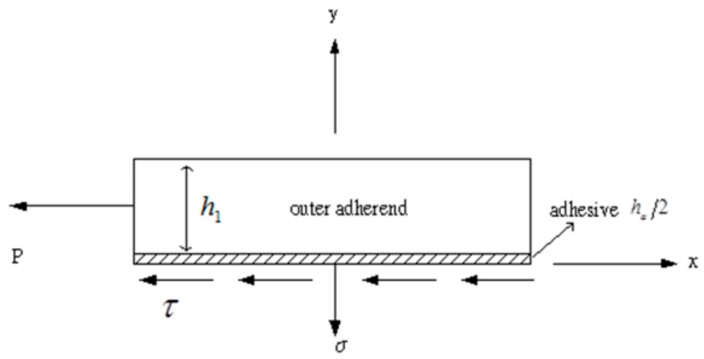
Free body diagram of the overlap region in the outer adherend and adhesive layer.

It is necessary to express these boundary conditions in terms of the shear stress τ and peel stress σ so that the constants $K_{1}$~$K_{7}$ can be determined. Figure 4 shows the free body diagram of the overlap region in the outer adherend and adhesive layer. The boundary conditions can be rewritten as

(30) $$\int_{-l}^{l}\tau \;dx=-P$$

(31) $$\int_{-l}^{l}\sigma \;dx=0$$

(32) $$\int_{-l}^{l}\sigma \;xdx-\frac{P(h_{1}+h_{a})}{2}=0$$

Substituting Equation (7a) into Equation (8a), leads to

(33) $$\sigma =\frac{E_{a}}{(1-\nu_{a}^{2})h_{a}}(w_{1}-w_{2})$$

Taking the derivative with respect to x twice for the above equation then substituting into Equation (4), yields

(34) $$\frac{d^{2}\sigma }{dx^{2}}=\frac{E_{a}}{(1-\nu_{a}^{2})h_{a}}(\frac{d^{2}w_{1}}{dx^{2}}-\frac{d^{2}w_{2}}{dx^{2}})=\frac{E_{a}^{\prime }}{h_{a}}(\frac{12}{E_{2}^{\prime }h_{2}^{3}}M_{2}-\frac{12}{E_{1}^{\prime }h_{1}^{3}}M_{1})$$

Substituting Equations (21), (28) and (29) into Equation (34), the boundary conditions can be rewritten in terms of the peel stress as follows

(35) $${\frac{d^{2}\sigma }{dx^{2}}|}_{x=l}=0$$

(36) $${\frac{d^{2}\sigma }{dx^{2}}|}_{x=-l}=0$$

Substituting Equation (6) into Equation (7b) and combining with Equation (8b), leads to

(37) $$\tau =\frac{G_{a}}{h_{a}}(u_{a}^{top}-u_{a}^{bottom})=\frac{G_{a}}{h_{a}}(u_{1}-u_{2}+\frac{1}{2}h_{1}\frac{dw_{1}}{dx}+\frac{1}{2}h_{2}\frac{dw_{2}}{dx})$$

Taking the derivative with respect to x for the above equation, yields

(38) $$\frac{d\tau }{dx}=\frac{G_{a}}{h_{a}}(\frac{du_{1}}{dx}-\frac{du_{2}}{dx}+\frac{1}{2}h_{1}\frac{d^{2}w_{1}}{dx^{2}}+\frac{1}{2}h_{2}\frac{d^{2}w_{2}}{dx^{2}})$$

Substituting Equations (4) and (5) into the above equation, yields

(39) $$\frac{d\tau }{dx}=\frac{G_{a}}{h_{a}}[\frac{1-\nu_{1}^{2}}{E_{1}}\frac{N_{1}}{h_{1}}-\frac{1-\nu_{2}^{2}}{E_{2}}\frac{N_{2}}{h_{2}}-\frac{1-\nu_{1}^{2}}{E_{1}}\frac{6}{h_{1}^{2}}M_{1}-\frac{1-\nu_{2}^{2}}{E_{2}}\frac{6}{h_{2}^{2}}M_{2}]$$

Substituting Equations (20) (28) and (29) into Equation (39), the boundary conditions can be rewritten in terms of the shear stress as follows

(40) $${\frac{d\tau }{dx}|}_{x=l}=-\frac{G_{a}}{h_{a}}\frac{2P}{E_{2}^{\prime }h_{2}}$$

(41) $${\frac{d\tau }{dx}|}_{x=-l}=\frac{G_{a}}{h_{a}}\frac{P}{E_{1}^{\prime }h_{1}}$$

Thus, boundary conditions are expressed in terms of the shear stress and peel stress as shown in Equations (30)–(32), (35), (36), (40) and (41). These equations can be used to determine the constants $K_{1}$~$K_{7}$.

## 4. Verification

The analytical solutions derived in the previous sections are validated with the results reported by Wu and Crocombe [34] and numerical solutions using the commercial finite element software ANSYS. In the validation problem, the material properties and thickness of the double lap joint are listed in Table 1. The longitudinal forces exerted on the outer and inner adherend are 200 N and 400 N, respectively. The bonding length is equal to 2l=18 mm.

Substituting the material properties and thickness listed in Table 1 into Equation (19), leads to the roots of the characteristic equations as follows

(42) $$m_{2}=\pm 0.39108;\;m_{3}=\pm 0.61341\pm 0.55362\;i$$

Substituting Equation (42) into Equations (20) and (21) then using the boundary conditions of Equations (30)–(32), (35), (36), (40) and (41), leads to the determination of the constants $K_{1}$~$K_{7}$ as follows

(43) $$\begin{array}{l} K_{1}=-1.6777\times 10^{-15};\;K_{2}=-8.7604\times 10^{-1};\;K_{3}=-2.6381;\;\\ K_{4}=-8.1861\times 10^{-3};\;K_{5}=-8.9682\times 10^{-2};\;K_{6}=-2.5075\times 10^{-2};\;K_{7}=-2.9412\times 10^{-2} \end{array}$$

Substituting the constants from Equations (42) and (43) into Equations (20) and (21), results in the shear and peel stresses in the adhesive as follows

(44a) $$\begin{array}{l} \tau (x)=-1.677\times 10^{-15}-8.7604\times 10^{-1}\sinh(0.39108x)-2.6381\times \cosh(0.39108x)\\ -8.1861\times 10^{-3}\sinh(0.61341x)\cos(0.55362x)-8.9682\times 10^{-2}\sinh(0.61341x)\sin(0.55362x)\\ -2.5075\times 10^{-2}\cosh(0.61341x)\cos(0.55362x)-2.9412\times 10^{-2}\cosh(0.61341x)\sin(0.55362x) \end{array}$$

(44b) $$\begin{array}{l} \sigma (x)=3.136\times 10^{-2}\cosh(0.39108x)+9.4438\times 10^{-2}\sinh(0.39108x)\\ +1.032\times 10^{-1}\cosh(0.61341x)\cos(0.55362x)-2.1201\times 10^{-1}\sinh(0.61341x)\sin(0.55362x)\\ -6.4703\times 10^{-1}\cosh(0.61341x)\sin(0.55362x)+3.1401\times 10^{-1}\sinh(0.61341x)\cos(0.55362x) \end{array}$$

**Figure 5:**
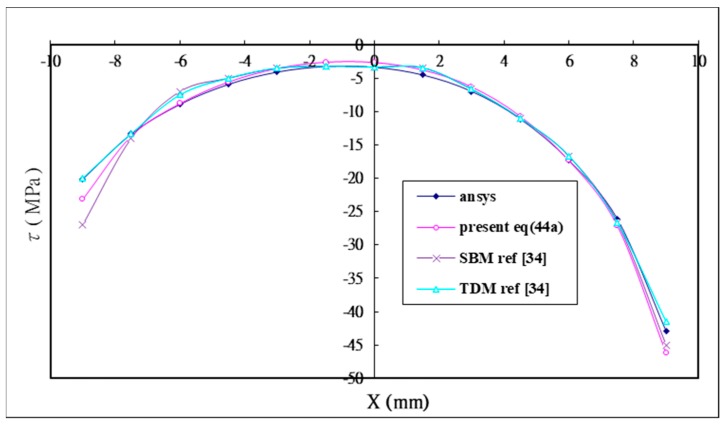
Analytical solution Equation (44a) of the shear stress in the adhesive compared with the numerical solutions using finite element software ANSYS and Wu [34].

**Figure 6:**
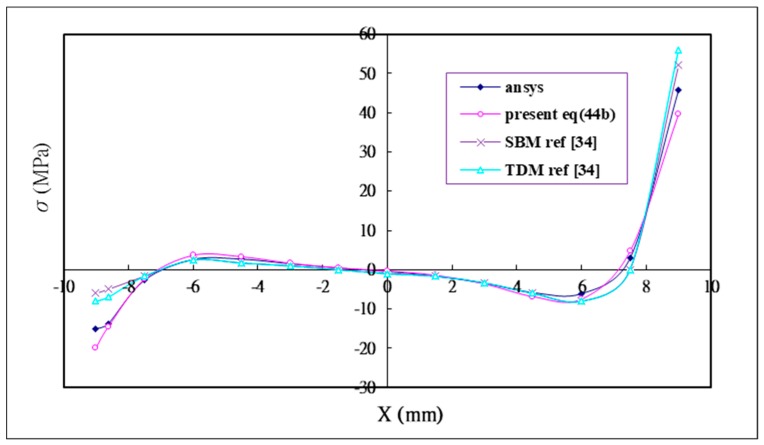
Analytical solution Equation (44b) of the peel stress in the adhesive compared with the numerical solutions using finite element software ANSYS and Wu [34].

In addition to the analytical solution, the finite element software ANSYS is performed to calculate the shear and peel stress in the adhesive for the validation problem. Wu and Crocombe [34] also solved the validation problem using two different finite element models, namely, simplified beam modeling (SBM), which modeled the adhesive layer as beam elements; and two dimensional modeling (TDM) which modeled the adhesive layer as two dimensional elements. The analytical solutions of the shear stress Equation (44a) and peel stress Equation (44b) are compared with the numerical results using ANSYS and two finite element models SBM and TDM proposed by Wu and Crocombe [34]. The shear and peel stress distribution in the adhesive are plotted in Figure 5 and Figure 6, respectively. It demonstrates that there are close agreements between the analytical solutions and numerical results.

## 5. Parametric Study

It is well known that the high shear and peel stresses at the edge of the bonding region are the most responsible for the failure of the adhesively bonding joints [17,35]. In this section, the parameters that affect the interfacial stress distributions in the adhesive are divided into two categories, material parameters and geometric parameters, respectively. Material parameters include the Young’s moduli of the adherends and the adhesive. Geometric parameters include the thickness of the adhesive and the length of the bonding region. In the following parametric study, the longitudinal forces exterted on the outer and inner adherends are 300 N and 600 N, respectively, while the material properties, thickness, and bonding length of the double lap joint are listed in Table 2. The adhesive used in this work is an epoxy.

### 5.1. The Effect of the Thickness of the Adhesive

**Figure 7:**
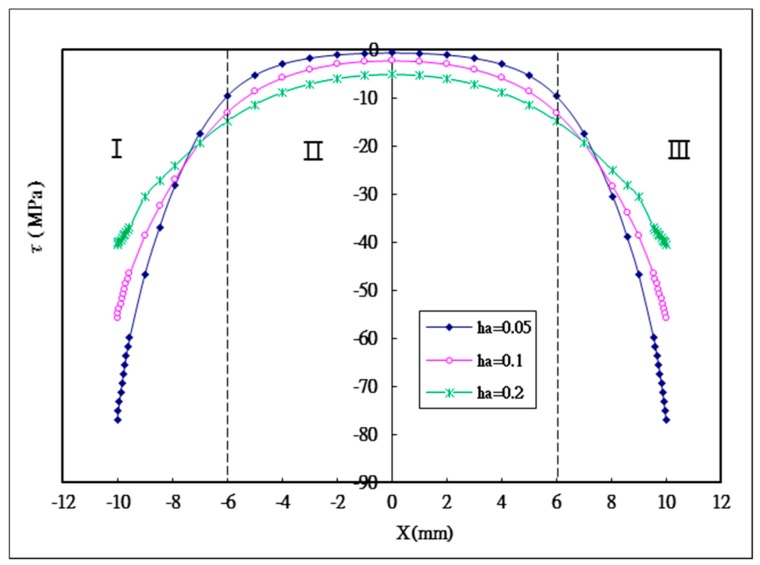
Shear stress distribution in the adhesive with three different adhesive thicknesses.

**Figure 8:**
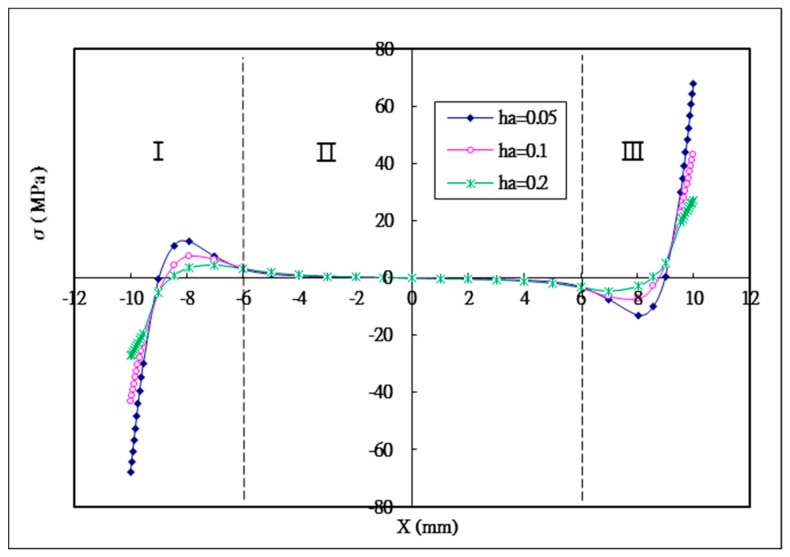
Peel stress distribution in the adhesive with three different adhesive thicknesses.

Three different adhesive thicknesses $h_{a}=$ 0.2 mm, 0.1 mm, 0.05 mm are considered in this study. The shear and peel stress distributions in the adhesively bonded region for the three different adhesive thicknesses are plotted in Figure 7 and Figure 8, respectively. It can be seen that shear stress appears in a symmetric distributuion, while peel stress exhibits an anti-symmetric distribution with respect to the middle point of the bonding region. The maximum shear and peeling stresses varying with the adhesive thickness obtained by Equation (44) and ANSYS are listed in Table 3. A reasonable agreement is achieved between the present approach and ANSYS finite element results. Based on the stress distribution, the bonding region can be divided in to three sections as shown in Figure 7 and Figure 8. In sections I and III, high stress level and gradient are observed due to the free edge effect [36,37]. In section II, lower stress and more even distribution appeared. The maximum shear stress that occured at the bonding ends is decreasing with the increase of the adhesive thickness. The maximum shear stress for the adhesive thickness of 0.05 mm is −77 Mpa. While, the maximum shear stress for adhesive thicknesses of 0.1 mm and 0.2 mm are −55.7 MPa and −40.4 MPa, respectively, representing reductions of 27.7% and 47.5%, respectively, in comparison with the adhesive thickness of 0.05 mm. The average shear stress in section II is increasing with the increase of the adhesive thickness. For the adhesive thickness of 0.05 mm, the average shear stress is −3.33 Mpa. The average shear stresses for adhesive thicknesses of 0.1 mm and 0.2 mm are −5.87 Mpa and −8.59 MPa, respectively, representing increases of 76.3% and 158%, respectively, in comparison with the adhesive thickness of 0.05 mm. This means that more shear stress can be transferred from the bonding ends to the middle region by increasing the adhesive thickness, resulting in a reduction of the stress concentration at the bonding ends.

Peel stress is more critical to the adhesive lap joint compared with shear stress, since it is considered to dominate the failure of the joint [26]. The maximum peel stress is negative in section I, representing that the left end of the bonding region is subjected to a compressive load. In section III, the maximum peel stress is positive, which means that the right end of the bonding region is under tension. In section II, peeling stress changes from positive to negative in the middle of bonding region, which means that a bending moment acts on this region. Similar behavior was also reported by Hou [26]. The peak value of the peeling stress is decreasing with the increase of the adhesive thickness. The maximum peeling stress for the adhesive thickness of 0.05 mm is 68 Mpa. While, the maximum peeling stress for adhesive thicknesses of 0.1 mm and 0.2 mm are 43 MPa and 27 MPa, respectively, representing reductions of 36.8% and 60.3%, respectively, in comparison with the adhesive thickness of 0.05 mm.

### 5.2. The Effect of the Young’s Modulus of the Adhesive

**Figure 9:**
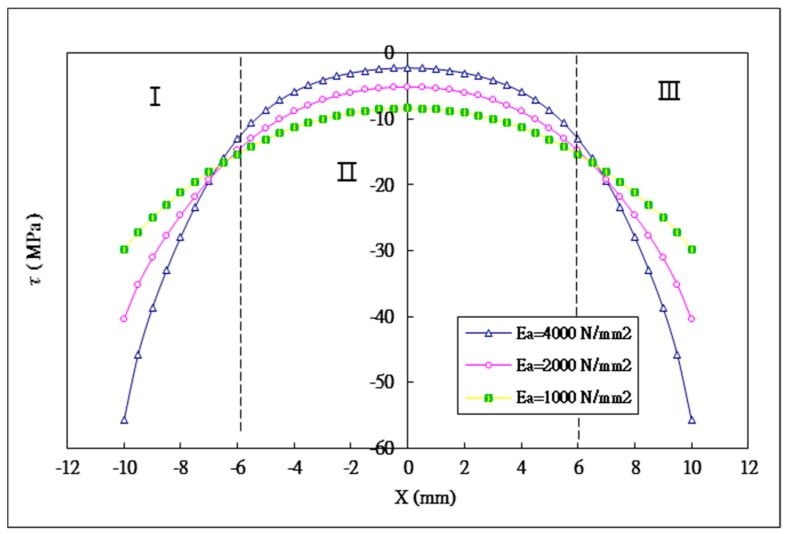
Shear stress distribution in the adhesive with three different Young’s moduli of the adhesive.

**Figure 10:**
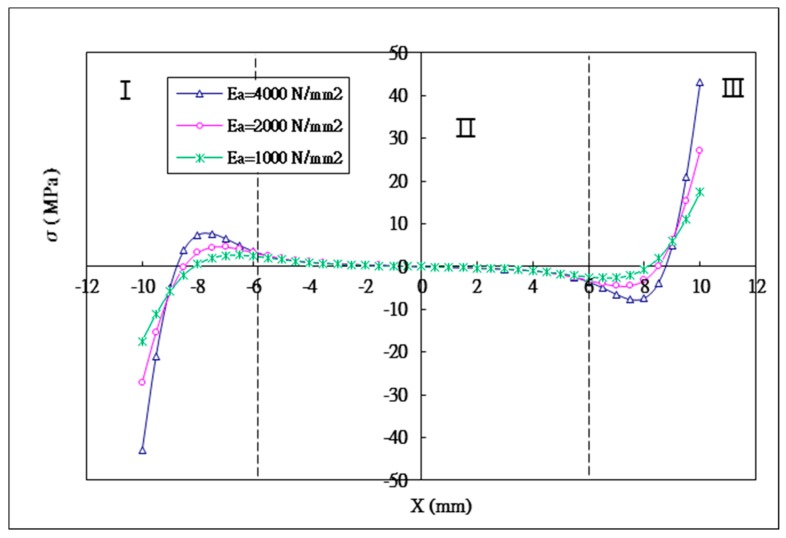
Peel stress distribution in the adhesive with three different Young’s moduli of the adhesive.

Three different Young’s moduli of the adhesive $E_{a}=$ 1 GPa, 2 GPa, 4 GPa are considered in this study. The effect of varying adhesive Young’s modulus on the shear and peel stress distributions in the adhesively bonded region are plotted in Figure 9 and Figure 10, respectively. The maximum shear and peeling stresses varying with the adhesive Young’s modulus obtained by Equation (44) and ANSYS are listed in Table 4. A reasonable agreement is achieved between the present approach and ANSYS finite element results. The maximum shear stresses occured at both ends of the bonding region for the adhesive Young’s moduli of 1 GPa, 2 GPa and 4 GPa are −29.9 MPa, −40.4 MPa and −55.7 MPa, respectively. The maximum shear stress is increasing with the increase of the adhesive Young’s modulus. Compared to the adhesive Young’s modulus of 1 GPa, the increase of the maximum shear stress for the adhesive Young’s moduli of 2 GPa and 4 GPa are 35.1% and 86.3%, respectively. In section II, the average shear stress for the adhesive Young’s moduli of 1 GPa, 2 GPa and 4 GPa are −10.8 MPa, −8.3 MPa and −5.8 MPa, respectively. The average shear stress is decreasing with the increase of the adhesive Young’s modulus. Compared to the adhesive Young’s modulus of 1 GPa, the reductions of the average shear stress for the adhesive Young’s moduli of 2 GPa and 4 GPa are 23.1% and 46.3%, respectively. The shear stresses are distributed more uniformly in the middle region (Section 2) as the adhesive Young’s modulus is decreasing.

The maximum peeling stresses that occured at both ends of the bonding region have the same magnitude but opposite sign. Figure 10 indicates that the magnitude of the peeling stress increases with the increasing adhesive Young’s modulus. The maximum peeling stresses for the adhesive Young’s moduli of 1 GPa, 2 GPa and 4 GPa are 17.4 MPa, 27.1 MPa and 43.1 MPa, respectively. Compared to the adhesive Young’s modulus of 1 GPa, the increase of the maximum peeling stress for the adhesive Young’s moduli of 2 GPa and 4 GPa are 55.7% and 147.7%, respectively. It can be observed that the adhesive stiffness has a large effect on the stress distribution, in particular the influence of the adhesive Young’s modulus on the peeling stress is more significant than that of the shear stress. A ductile adhesive is able to redistribute the load due to a plastic deformation, results in a higher average stress in the middle region and reduction of the stress concentration at the bonding ends. For a brittle adhesive, most of the loads are carried on the ends of the bonding region leading to a high stress concentration [2].

### 5.3. The Effect of the Bonding Length

**Figure 11:**
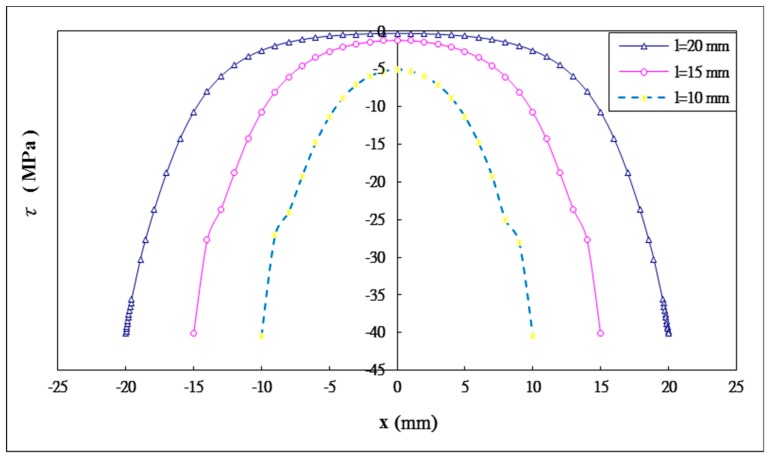
Shear stress distribution in the adhesive with three different bonding lengths.

**Figure 12:**
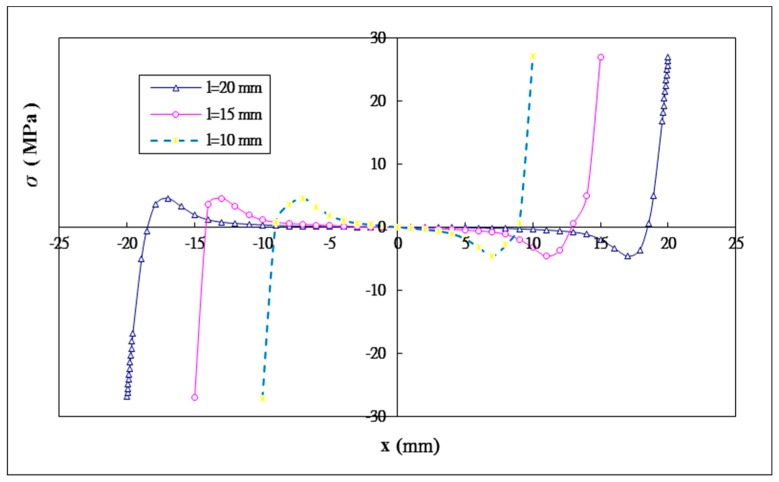
Peel stress distribution in the adhesive with three different bonding lengths.

Three different bonding lengths 2l= 20 mm, 30 mm, 40 mm are considered in this study. The influence of the bonding length on the shear and peel stress distributions in the adhesive are plotted in Figure 11 and Figure 12, respectively. The maximum shear and peeling stresses varying with the bonding length obtained by Equation (44) and ANSYS are listed in Table 5. A reasonable agreement is achieved between the present approach and ANSYS finite element results. The maximum shear stresses for bonding lengths of 20 mm, 30 mm and 40 mm are −40.13 MPa, −40.15 MPa, and −40.41 MPa, respectively. The maximum peeling stresses for bonding lengths of 20 mm, 30 mm and 40 mm are 26.93 MPa, 26.94 MPa, and 27.14 MPa, respectively. It appears that the maximum values of shear and peel stresses increase silghtly when the bonding length increases. Similiar results have been reported by Names and Lachaud [38]. They found that increasing the bonding length over a certain value does not have any significant effect on the maximum stress in the adhesive. In fact, for a double lap joint there is an optimal bonding length beyond which the added length is not carrying loads.

### 5.4. The Effect of the Young’s Modulus of the Inner Adherend

**Figure 13:**
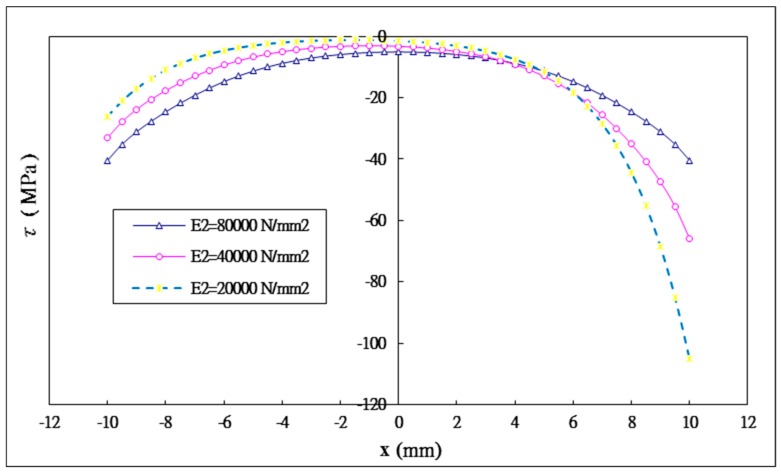
Shear stress distribution in the adhesive with three different Young’s moduli of the inner adherend.

**Figure 14:**
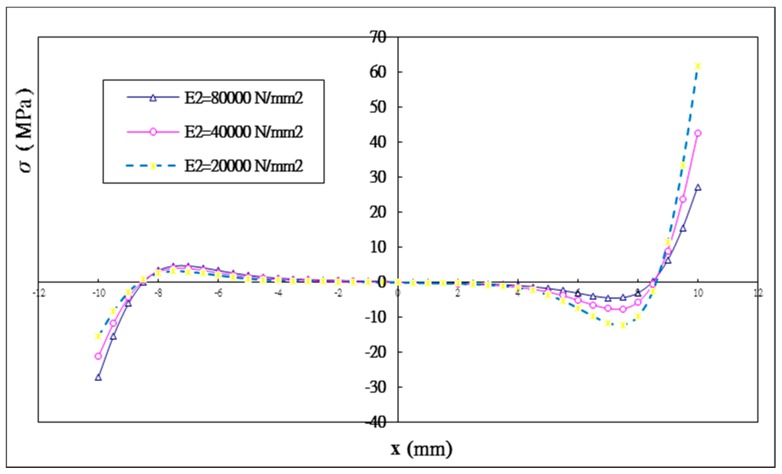
Peel stress distribution in the adhesive with three different Young’s moduli of the inner adherend.

Three different Young’s moduli of the inner adherend $E_{2}=$ 80 GPa, 40 GPa, 20 GPa are considered while the Young’s modulus of the outer adherend is kept at a constant of 80 GPa. The shear and peel stress distributions in the adhesively bonded region for these three different Young’s moduli of the inner adherend are plotted in Figure 13 and Figure 14, respectively. The maximum shear and peeling stresses varying with the inner adherend Young’s modulus obtained by Equation (44) and ANSYS are listed in Table 6. A reasonable agreement is achieved between the present approach and ANSYS finite element results. It can be seen that the shear stress distribution is no longer symmetric and the peel stress distribution is no longer anti-symmetric as the material properties of the inner and outer adherends are different. As the Young’s modulus of the inner adherend decreases from 80 GPa to 20 GPa, the maximum shear stress at the right end of the bonding region is increasing from −40.4 MPa to −105.1 MPa, while the maximum shear stress at the left end of bonding region is decreasing from −40.4 MPa to −26.0 MPa. For the peeling stress, the maximum value at the right end of the bonding region is increasing from 27.1 MPa to 59.4 MPa, while the maximum value at the left end of the bonding region is decreasing from −27.1 MPa to −15.5 MPa. A similar behavior was observed by Diaz [39] for heterogeneous double-lap joints.

Apparently, the interfacial stresses distributions are significantly affected by the stiffnesses of both the inner and outer adherends. The right end of the bonding region is located at the free end of the outer adherend. The higher the stiffness of the outer adherend relative to the inner adherend the more loads are transferred to the outer adherend, resulting in a larger stress concentration at the right end of the bonding region due to the edge effect of the outer adherend. The maximum shear stresses at the right end of the bonding region for the inner adherend Young’s moduli of 40 GPa and 20 GPa are increased by 63.1% and 160%, respectively, in comparison with the Young’s modulus of 80 GPa. The maximum peeling stresses at the right end of the bonding region for the inner adherend Young’s moduli of 40 GPa and 20 GPa are increased by 56.1% and 119.2%, respectively. The left end of the bonding region is located at the free end of the inner adherend. The higher the stiffness of the inner adherend the more loads are carried by the inner adherend, leading to a larger stress concentration at the left end of the bonding region due to the edge effect of the inner adherend. The maximum shear stresses at the left end of the bonding region for the inner adherend Young’s moduli of 40 GPa and 20 GPa are decreased by 18.1% and 35.6%, respectively, in comparison with the Young’s modulus of 80 GPa. The maximum peeling stresses at the left end of the bonding region for the inner adherend Young’s moduli of 40 GPa and 20 GPa are decreased by 21.7% and 42.8%, respectively.

## 6. Discussion

**Figure 15:**
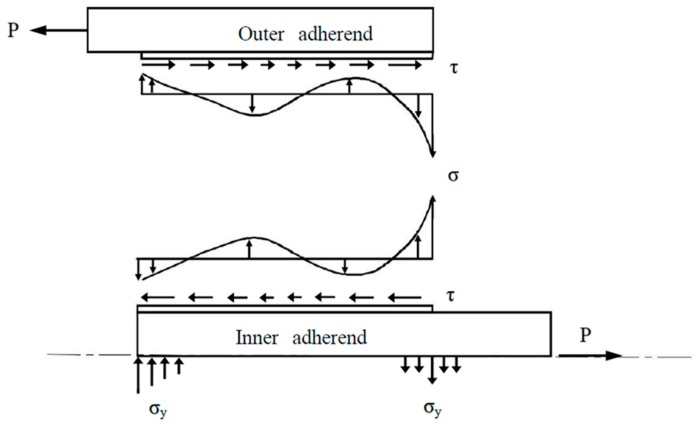
Schematic diagram of the interfacial stress distribution in the adhesive.

Interfacial stresses in the adhesive are critical to the prediction of the failure of the double lap joint. In this work, the interfacial stresses including the shear and peeling stresses are derived analytically. Based on the numerical results from the previous section, the interfacial stress distribution in the adhesive is schematically illustrated in Figure 15. It is noted that the peeling stress changes sign along the interface resulting in a bending affect on the joint. It also found that a tensile peeling stress is exerted on the right end of the bonding region, while a compressive peeling stress is applied on the left end. Both the shear and peeling stresses are increasing rapidly at the ends of the bonding region due to the free edge effect. The high stress concentration at the ends of the bonding region may lead to the initiation of the joint failure. It is reported by Yousefsani [40] that the influence of the free edge effect on the interfacial stresses is confined in the region near to the bonding ends with the length approximate to the adhesive thickness. Numerical simulation results show that more uniform interfacial stress distribution can be achieved by increasing the adhesive thickness. The peak values of the shear and peeling stresses are decreasing with the increase of the adhesive thickness. A possible explanation of this observation is that a thicker adhesive is less susceptible to deformation than a thinner one. Adam and Peppiatt [41] found that the maximum stress is proportional to the reciprocal of the square root of the adhesive thickness provided the adhesive thickness is sufficiently small. Thus, it can be expected that the strength of the double lap joint is enhanced by the increase of the adhesive thickness. The adhesive stiffness also exhibits a large effect on the stress distribution in the adhesive. A ductile adhesive with a lower Young’s modulus leads to a more uniform stress distribution in the middle region and a less stress concentration at the bonding ends. A brittle adhesive with a larger Young’s modulus carries most of the loads at the ends of the bonding region, resulting in a higher stress concentration at the adhesively bonded ends and a reduction of the joint strength. In view of these results, Campilho and Fernandes [2] recommended that a less strong but ductile adhesive is the better choice. In the bonding region, the loads carried by the outer or inner adherend are dependent on their stiffness, respectively. The higher the stiffness the more loads are transferred to the adherend. The right end of the bonding region is located at the free edge of the outer adherend. The stress concentration at the right end of the bonding region is significantly affected by the load exerted on the outer adherend. Thus, the stress concentration at the right end of the bonding region is increasing with the increase of the Young’s modulus of the outer adherend owing to the more loads carried by the outer adherend. In contrast, the left end of the bonding region is located at the free edge of the inner adherend. The stress concentration at the left end of the bonding region is governed by the stiffness of the inner adherend, and increasing with the increase of the Young’s modulus. It should be noted that the results presented in this work are based on the assumption of a small adhesive thickness in comparison with that of adherends. Thus, interfacial stresses are constant across the adhesive thickness.

## 7. Conclusions

In this work, analytical solutions of the shear and peel stresses in the adhesive for adhesively bonded double lap joints are developed using Euler’s beam theory and elasticity theory. The distributions of interfacial peel and shear stresses are determined for the double lap joint subjected to a longitudinal force. The accuracy and effectiveness of the present theory in describing the stress state are illustrated by comparing the analytical solutions with the outcomes available in the literature as well as with those obtained by the finite element method. The effects of the adhesive thickness, bonding length and the Young’s moduli of the adhesive and adherends are investigated through a parametric study. The results demonstrate significant changes in the magnitude of interfacial stress components near the edges of the bonding region. Numerical results show that the thicker the adhesive thickness the less stress concentration will occur at the bonding ends. The maximum shear and peeling stresses are decreased by 47.5% and 60.3%, respectively, as the adhesive thickness increases from 0.05 mm to 0.2 mm. Both the maximum shear and peel stresses can be increased with the increase of the Young’s modulus of the adhesive. Parametric study shows that the maximum shear and peeling stresses are increased by 86.3% and 147.7%, respectively, as the adhesive Young’s modulus increases from 1 GPa to 4 GPa. In the case of two different adherends bonded by the adhesive, the larger the difference the greater the maximum shear and peel stresses will be induced at the right end of the bonding region. Numerical results show that the maximum shear and peeling stresses are increased by 160% and 119%, respectively, as the inner adherend Young’s modulus decreases from 80 GPa to 20 GPa while the outer adherend Young’s modulus is kept at a constant of 80 GPa.

## Figures and Tables

**Table 1:** Material properties and thickness of the double lap joint for the validation problem.

	Outer Adherend	Inner Adherend	Adhesive
Young’s modulus	70 GPa	70 GPa	2.1 GPa
Poisson ratio	0.3	0.3	0.4
Thickness	2 mm	2 mm	0.1 mm

**Table 2:** Material properties, thickness and bonding length of the double lap joint for the parametric study.

	Outer Adherend	Inner Adherend	Adhesive
Young’s modulus	80 GPa	80 GPa	2 GPa
Poisson ratio	0.3	0.3	0.4
Thickness	1 mm	2 mm	0.2 mm
Bonding length	20 mm	20 mm	20 mm

**Table 3:** Comparison of maximum interfacial stresses with different adhesive thicknesses obtained by Equation (44) and ANSYS.

Adhesive Thickness	Maximum Shear Stress (MPa)			Maximum Peeling Stress (MPa)		
	Equation (44a)	ANSYS	Difference	Equation (44b)	ANSYS	Difference
0.05 mm	−77.0	−70.4	8.57%	68.0	76.0	−11.76%
0.1 mm	−55.7	−54.1	2.87%	43.0	45.1	−4.88%
0.2 mm	−40.4	−39.1	3.22%	27.1	29.2	−7.75%

**Table 4:** Comparison of maximum interfacial stresses with different adhesive Young’s modulus obtained by Equation (44) and ANSYS.

Adhesive Young’s Modulus	Maximum Shear Stress (MPa)			Maximum Peeling Stress (MPa)		
	Equation (44a)	ANSYS	Difference	Equation (44b)	ANSYS	Difference
1 GPa	−29.9	−29.8	0.33%	17.4	19.4	−11.49%
2 GPa	−40.4	−39.1	3.21%	27.1	29.2	−7.75%
4 GPa	−55.7	−51.6	7.36%	43.1	44.8	−3.94%

**Table 5:** Comparison of maximum interfacial stresses with different bonding length obtained by Equation (44) and ANSYS.

Bonding Length	Maximum Shear Stress (MPa)			Maximum Peeling Stress (MPa)		
	Equation (44a)	ANSYS	Difference	Equation (44b)	ANSYS	Difference
20 mm	−40.13	−38.8	3.31%	26.93	8.9	−7.32%
30 mm	−40.15	−38.8	3.36%	26.94	29.0	−7.65%
40 mm	−40.41	−39.1	3.24%	27.14	29.2	−7.59%

**Table 6:** Comparison of maximum interfacial stresses with different inner adherend Young’s modulus obtained by Equation (44) and ANSYS.

Inner Adherend Young’s Modulus	Maximum Shear Stress (MPa)			Maximum Peeling Stress (MPa)		
	Equation (44a)	ANSYS	Difference	Equation (44b)	ANSYS	Difference
20 GPa	−105.1	−95.1	9.51%	59.4	61.7	−3.87%
40 GPa	−65.9	−62.7	4.86%	42.3	43.8	−3.55%
80 GPa	−40.4	−39.1	3.22%	27.1	29.2	−7.75%
